# Medication non-adherence and associated factors among diabetes patients in Felege Hiwot Referral Hospital, Bahir Dar city administration, Northwest Ethiopia

**DOI:** 10.1186/s13104-019-4205-4

**Published:** 2019-03-27

**Authors:** Teshager W/giorgis Abate

**Affiliations:** Department of Nursing, College of Medicine and Health Science, Bahir Dar University, P.O.BOX:79, Bahir Dar, Ethiopia

**Keywords:** Medication non-adherence, Adult patient with diabetes mellitus, Non-adherent factors

## Abstract

**Objective:**

This study was aimed to assess medication non-adherence and associated factors among adult diabetes in Felege Hiwot Referral Hospital Bahir Dar city administration. To overcome this object, a cross-sectional study was conducted among 416 randomly selected diabetes patients at the Felege Hiwot Referral Hospital (FHRH). Eight item Morisky Medication Adherence Scale questionnaire was used to assess medication non-adherence. Binary logistic regression was applied to analyze the collected data. P-value less than 0.05 with 95% confidence interval was considered statistically significant between dependent and explanatory variables.

**Result:**

Among 416 participants, 242 (58.2%) were male diabetes patient. The mean age (± SD) of the study participant was 45.4 (± 16. 7) years. Based on the MMAS-8 scale, non-adherence to diabetes medication was 68.8% [95% CI 62.0, 71.4]. The multivariate analysis, age group from 18 to 35 years old (AOR: 2.26: 95% CI 1.23, 5.58), single (AOR: 3.55; 95% CI 1.59, 7.29), fear of diabetes related complication (AOR: 3.01; 95% CI 1.66, 5.53) and feeling worse (AOR: 2.55; 95% CI 1.45, 4.53) were significantly associated with non-adherence to prescribed diabetes medications. Therefore, developing a more intensive communication strategy and improving the quality of prescribed drug compliance could improve the level of adherence.

## Introduction

Globally, Diabetes mellitus (DM) is increased from 424.9 million in 2017 to 628.6 million by the year 2045. It is a high level in sub-Saharan Africa [1–3]. In Ethiopian, the prevalence ranged from 0.3 to 7% [4–6]. Medication adherence to DM treatment is the extent to which the patient’s behavior matches the agreed recommendations from the prescriber. It is an active role, collaboration with the physician with no place for blame; self-motivated decision to adhere the advice and understood self-regulation [6, 7]. Medication non-adherence is dose not taken, irregular dosing and discontinuation medication [8, 9].

In Ethiopia, the proportion of non-adherence was ranged from 21.8 to 25.4% [10–12]. A previous study documented the proportion of non-adherence was shown different results in different setting. These were: Sudan (55%) [13]; Asia (21.9%) [14]; Switzerland (80%) [15]; Botswana (41.8%) [16]; Nigeria (73.64–86.6%) [17, 18] and Ghana (31.5%) [19] of participants were non-adherent to diabetes treatment. Poor health status, service dissatisfaction [11, 15]; educational level [14, 20]; age, gender and comorbidity [15, 18] were factors affecting medication non-adherence.

Non-adherence also affected by diabetes knowledge, disease duration [20]; perception of consequence [14, 21]; psychological problems and forgetfulness [7, 12, 13, 22, 23]. Thus, non-adhere to medication increased emergency room visits and hospitalization [24]. Studies on medication non-adherence in diabetes patient were limited in the study area. Therefore, the aim of this study was to identify medication non-adherence and associated factors among DM patient.

## Main text

### Study setting and participants

This was a cross sectional study with patients recruited from February 21st to March 21st 2017 at FHRH. The hospital provides promotive, preventive, curative and rehabilitative services. Around 2484 diabetes patients were registered for follow-up in the previous year. In outpatient chronic follow up department approximately 250 adult DM patients were seen weekly. Patients with DM were visited the hospital on every 2 months basis.

The sample size was determined using a single population formula by considering 45.9% proportional of adherence to diabetes medication [11]; 95% confidence interval (CI), 5% margin error as $n=\frac{(z {\frac \alpha2})^2\times p(1-p)}{d^{2} }$. After adding 10% non-response rate yielding 420 DM patients.

Systematic random sampling was employed to select eligible participants. Based on the decision to collect data over the course of 1 month, sampling interval was determined by dividing the expected number of diabetic patients per month (1000) into the sample size (420). Thus, every other patient coming to a follow-up service was interviewed in daily basis. A patient with both types of diabetes aged above 18 years and who have been taking diabetes medication in the last 6 months in a regular follow up was included.

Trained four BSc nurses collected data through face to-face interview and medical chart review. The questionnaire contains socio-demographic, clinical and other related factors: social support assessed by Oslo 3-items social support scale. The sum of this scale was ranging from 3 to 14. A scale ranged from 3 to 8 was categorised as “poor”, 9–11 was “moderate” and 12–14 was “strong” support [25].

Fear of complication was assessed by fear of complications questionnaires. It includes specific fears (like blindness, kidney problems, etc.), lifestyle, and hypoglycemia fears. The items were rated on a four-point Likert scale (0–3), where “0” denotes low and “3” refers to high fear of complications [26]. The anxiety level was determined by a four-point Likert scale (0–3) of the anxiety Sub scale of Hospital Anxiety and Depression Scale, where “0” denotes non-anxiety symptom and “3” refers had anxiety symptoms. Scores of 0–7 was considered normal, 8–10 borderline and 11–21 indicating clinical cases (abnormal) [27]. In this study, borderline was considered as normal.

Self-reported adherence to diabetes medications was determined by MMAS-8. All questions, except for the last question, were answered with a yes/no response, with corresponding 1 and 0 value [28]. Zero score was considered high adherence, 1or 2 as medium adherence and > 2 was low adherence. In this study, medium and high adherences were considered as adherence and low adherence as non-adherent for statistical purpose [29, 30].

Structured questionnaire was prepared in English. It was translated into local language ‘Amharic’ to easily understand and then back translated into English to check the consistency. The questionnaire was pre-tested among 21 DM patients at Debre-Tabor hospital. Minor modification like ambiguity words was edited. The principal investigator and two supervisors were responsible monitoring the data collection process.

The collected data entered into the Epi-data version 3.1 and analyzed using SPSS version 20. Descriptive and summary statistics were presented using texts and tables. Binary logistic regression analysis was performed to determine the association between dependent and independent variables. Independent variables with a P-value < 0.2 in the bi-variable analysis were fitted into multivariate analysis to identify independent predictors of non-adherence. Associated factors were expressed as Adjusted OR with 95% CI and P-value < 0.05 was considered as statically significant.

### Result

A total of 416 participants completed the interview giving a response rate of 99.05%. Of these, 242 (58.2%) were male diabetes patient. The mean age (± SD) of the study participant was 45.4 (± 16. 7) years (Table 1).

**Table 1:** Socio-demographic characteristics of study participant at FHRH Bahir Dar, Northwest Ethiopia, (n = 416)

Variable	Frequency	Percent
Sex		
Female	174	41.8
Male	242	58.2
Age		
18–36	141	33.9
36–50	122	29.3
51–65	89	21.4
66–80	64	15.4
Residency		
Rural	100	24.0
Urban	316	76.0
Marital status		
Divorced	17	4.1
Single	32	7.7
Separated	88	21.2
Married	279	67.1
Educational qualification		
Never went	133	32.0
Primary school	142	34.1
Secondary school	55	13.2
Tertiary	86	20.7
Occupational status		
Farmer	147	35.3
Civil servant	130	31.3
Private worker	47	11.3
Merchant	92	22.1
Average monthly income		
≤ 500	94	22.6
501–1000	107	25.7
1001–2000	88	21.2
≥ 2001	127	30.5

Two hundred fifty (60.1%) participants were type 2 DM. The mean duration since diagnosed with diabetes was 6 (± 4.9) years. About 26% had a fear of complication due to DM (Table 2).

**Table 2:** Clinical and psychosocial characteristics of study participant at FHRH, Bahir Dar, Northwest Ethiopia, (n = 416)

Variable	Frequency	Percent
DM types		
Type 1	166	41.9
Type 2	250	60.1
DM duration		
≤ 5	242	58.2
6–10	113	27.2
≥ 11	61	14.7
DM comorbid		
Yes	100	24.0
No	316	76.0
Fasting blood glucose (mg/dL)		
≤ 91	65	15.6
91–126	106	25.5
127–2000	145	34.9
≤ 201	100	24.0
DM complication		
Yes	85	20.4
No	331	79.6
Social support		
Poor	71	17.1
Moderate	132	31.7
Strong	213	51.2
Fear of complication		
High fear	108	26.0
Low fear	308	74.0
Anxiety		
Abnormal	107	25.8
Normal	309	74.2

Self-reported non-adherence to diabetes medications, based on MMAS-8, was 68.8% [95% CI 62.0, 71.4]. The multivariate logistic regression analysis showed that participant who were living in rural (AOR: 2.35; 95% CI 1.25, 3.23), being single (AOR: 3.55; 95% CI 1.59, 7.29), merchant (AOR: 3.32; 95% CI 1.22, 9.02), a high fear of complication (AOR: 3.01; 95% CI 1.66, 5.53) and feeling worse (AOR: 2.55; 95% CI 1.45, 4.53) were more likely non-adhere to diabetes medications (Table 3).

**Table 3:** Factors associated with diabetes medication non-adherence among diabetes at FHRH, Bahir Dar, Northwest Ethiopia, (n = 416)

Variable	Adherence level		Bivariate	Multivariate
	Adherence (n)	Non-adherence (n)	Crude OR (95% CI)	Adjusted OR (95% CI)
Age				
< 36	64	77	2.71 [1.93,5.29]	2.62 [1.23, 5.58]
36–50	32	90	1.16 [0.57, 2.35]	1.30 [0.58, 2.95]
51–65	27	62	1.42 [0.68, 2.96]	1.38 [0.59, 3.23]
≥ 66	15	48	1	1
Residency				
Rural	221	57	1.76 [1.10, 2.79]	2.35 [1.25, 4.42]
Urban	95	43	1	1
Marital status				
Divorced	3	14	0.49 [0.14, 1.76]	0.62 [0.16, 2.38]
Single	20	12	3.87 [1.81, 8.27]	3.55 [1.59, 7.29]
Separated	31	57	1.26 [0.76, 2.09]	1.50 [0.87, 2.58]
Married	84	195	1	1
Occupational				
Private worker	6	41	1	1
Merchant	33	59	3.82 [1.49, 9.95]	3.32 [1.22, 9.02]
Farmer	51	96	3.63 [1.44, 9.12]	1.94 [0.71, 5.33]
Civil servant	48	82	4.00 [1.58, 10.12]	4.06 [1.54, 10.68]
Comp. fear				
High	56	52	2.96 [1.88, 4.67]	3.01 [1.66, 5.53]
Low	82	226	1	1
Anxiety				
Abnormal	98	110	3.74 [5.81]	2.55 [1.45, 4.53]
Normal	40	168	1	1

n = number, P ≤ 0.05 was taken as level of significance, OR = odd ratio

### Discussion

This study demonstrated that about 68.8% of the participants had non-adherence to diabetic medication. The finding of this study was comparable to a study conducted in Switzerland [13], Nigeria [17] and India [30, 31]. Perhaps this might be due to methodological similarity and use of the similar tool.

On the other hand, the proportion of non-adherence in our study was much higher than when compared with other similar studies done in Bharat [14], Botswana [16], Ghana [19], New York [29], Gondar [14] and Jimma [12]. This difference might be in those study participants, the health care provider simplifying a patient’s medication by providing written instructions and provides diabetes education about medication and patient’s behavior matches the agreed recommendations [32]. Another discrepancy might be due to sample size and study design difference.

In this study occupational statuses like merchant (3.32 times) and civil servant (4.06 times) more likely non-adherence when compared to private employees and farmers. This finding was congruent with a study conducted in Pakistan [33]. This similarity might be the greater one’s income, the lower one’s likelihood of non-adherence among private employees and sedentary lifestyle among merchants.

In our study, patients with age 18–35 years had 2.62 times more likely non-adherence when compared to with those aged 36–80 years. This finding was comparable to a study conducted in Pakistan and Switzerland [15, 34]. This might be, among younger age groups (18–35) might be to a lesser extent understanding of treatment recommended and adherence [35].

In our study, participants who were single in marital status were 3.55 times more likely non-adherence than as compared to married. A contrasting result was found in a study done in Portugal single individuals were better to medication adherence [22]. This might reflect different methodologies, sampled population and sample size. A study conducted in Portugal was correlational design, with a smaller sample size and aged 40 to 85 years. In our study non-adherence in single individuals might a lack of emotional and practical support from the spouse that leads to claimed to have contributed to non-adherence. This was due to perceiving non supportive behaviors were associated non-adherence [36, 37].

This study revealed that participants who had anxiety 2.55 times more likely non-adhere as compared to those who had not anxiety. This finding was in line with a study documented in Bharat, Portugal and Kingdom of Saudi Arabia [14, 22, 23]. This might be due to diabetes patients with anxiety had been at increased to feel guilt and low treatment outcome [38].

This study showed that place of residence was significantly associated with non-adherence. Those diabetes patients who live in rural were 2.35 times more likely non-adherence as compared to urban. This was in line with a study conducted in Bharat [14]. Individuals with rural residency might be not accessed transportation and drugs [39].

Fear of complication was another variable that significantly associated with non-adherence to prescribed medication. Fear of complication was 3.01 times more likely non-adherence when compared to those who had a low fear. This was consistent with a study done by different scholars [21, 32, 33] which stated that factors of non-adherence.

## Limitations

Even if the author was making a great effort to orientation and explain about the aim of the study, recall bias might creep. Since the study was conducted in a clinic setting, a social desirability bias might have made.

### Conclusion

We conclude that medication non-adherence is prevalent among DM patients and is associated with fear of complication, social support and anxiety. Therefore, this study recommends that the hospital facilitates assessment of medication non-adherence should be incorporated into routine clinical practice. Interventions are urgently needed to increase medication adherence so that patients can realize the full benefit of prescribed therapies.
